# Exploring sustainable synthesis paths: a comprehensive review of environmentally friendly methods for fabricating nanomaterials through green chemistry approaches

**DOI:** 10.55730/1300-0527.3691

**Published:** 2024-05-29

**Authors:** Vishu GIROTRA, Pritam KAUSHIK, Dipti VAYA

**Affiliations:** Department of Chemistry, Biochemistry, and Forensic Science, Amity School of Applied Science, Amity University, Haryana, Gurugram, India

**Keywords:** Microorganisms, green synthesis, plant extract, metal, metal oxide

## Abstract

This comprehensive review delves into the burgeoning field of nanotechnology, where the synthesis of nanoparticles (NPs) is strategically tailored to specific applications. Embracing the principles of green chemistry, nanotechnology increasingly utilizes environmentally friendly materials, such as plant extracts or microorganisms, as capping or reducing agents and solvents in the synthesis process. Notably, plant-based synthesis demonstrates enhanced stability and faster rates compared to microorganisms. The synthesized materials exhibit unique properties ranging from antimicrobial and catalytic effects to antioxidant activities and they are finding applications across diverse fields. Green synthesis processes, characterized by mild conditions in terms of temperature and reagents, stand in stark contrast to traditional chemical synthesis methods. This review focuses on the synthesis of various metal and metal oxide NPs, including Ag, Au, Zn, Fe, Mg, Ti, Sn, Cu, Cd, Ni, Co, and Ag NPs and their oxides, using plant extracts and microorganisms. We provide a comprehensive analysis of the advantages, disadvantages, and applications associated with each synthesis method. Additionally, we explore the future prospects of green synthesis and its limitations and challenges, offering insights into its evolving role in nanotechnology.

## 1. Introduction

In the past few years, the study of nanotechnology has grown exponentially, achieving new heights in various fields such as food technology, healthcare, optical devices, the space industry, cosmetics, water supply, and electronics [1–3]. Its incorporation in various research areas such as chemistry, engineering, physics, material science, and biology is being explored [4,5]. Nanomaterials, usually ≤100 nm in size, could exhibit outstanding chemical and physical properties in bulk due to their high specific surface areas [6,7].

**Figure 1:**
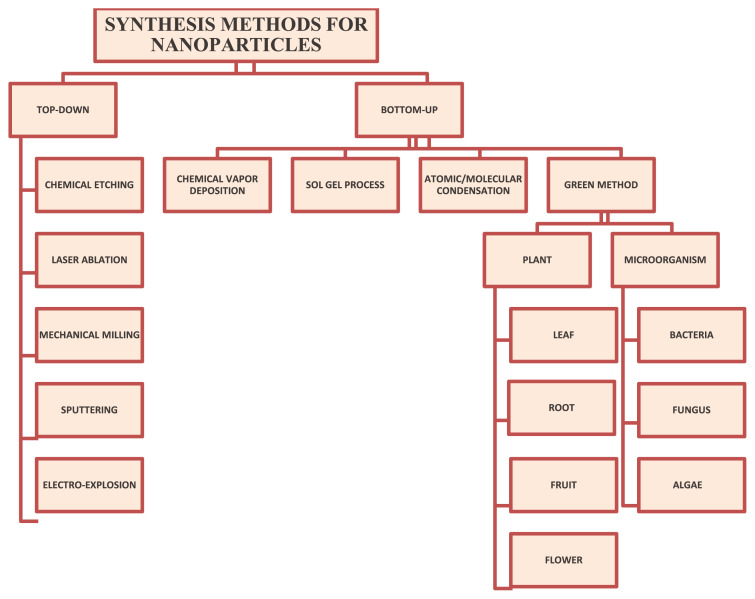
Synthesis methods for nanoparticles.

The two different main approaches for the synthesis of nanomaterials are bottom-up and top-down, as shown in Figure 1. In the top-down approach, bulk materials are broken down to the nanoscale, while in the bottom-up approach, molecules or atoms are assembled to form nanoparticles (NPs) [8–10]. A large number of methods have been reported for the synthesis of nanomaterials, including the coprecipitation method, hydrothermal method, sol-gel method, and sonochemical method [11]. These methods are highly expensive and toxic, raising problems of toxic waste generation and energy imbalance. Hence, the green eco-friendly synthesis of nanomaterials is attracting attention among researchers [12]. Green synthesis methods for nanomaterials constitute an eco-friendly, less expensive, clean, and relatively newer field of study [4,8,13].

Metal oxide nanomaterials such as TiO_2_, SnO_2_, and ZnO that offer good optical and electronic properties and can be used in supercapacitors have been widely studied [9–11]. Biogenic metallic nanomaterials could be produced by various organisms such as plants, bacteria, yeast, and actinomycetes. Some other microorganisms like fungi, which offer several advantages, such as high cell wall-binding capacity, the production of various extracellular enzymes, and high biomass capacity, are also used in green synthesis methods [14,15]. These biogenic nanomaterials are utilized in wastewater treatment and for their antimicrobial activity.

Wastewater effluents are generated by various industries, such as textile, paper, plastic, cosmetics, and pharmaceutical industries. These effluents largely consist of organic pollutants such as dyes and phenolics, which are toxic and carcinogenic in nature [16]. These dyes reduce the photosynthesis process of aquatic plants by blocking the full intensity of sunlight and reducing the oxygen-carrying capacity of water [17]. Many studies have reported on green synthesis with nickel, palladium, cobalt, tin, iron, gold, titania, zinc, silver, copper, cadmium, and magnesium [13,18,19]. This review addresses green synthesis approaches and describes various green sources used for the synthesis of transition metals and metal oxides. We also explore the advantages and disadvantages, limitations, and future directions for green synthesis methods.

## 2. Green synthesis

**Figure 2:**
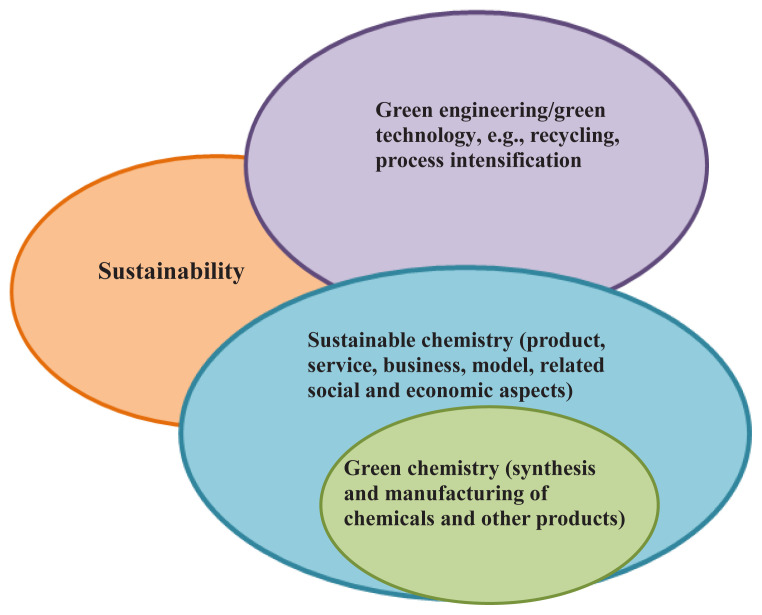
Green synthesis of NPs.

The term “green chemistry” was coined by Paul Anastas, who is considered as the father of green chemistry. It is defined as the invention, design, and application of chemical products to decrease or remove the use and generation of toxic substances. It creates new alternative paths allowing the use of less hazardous materials. Green synthesis approaches entail transformations of existing ideas and research efforts in the context of resolving the problems of chemical pollution and resource depletion, as shown in Figure 2 [15].

**Figure 3:**
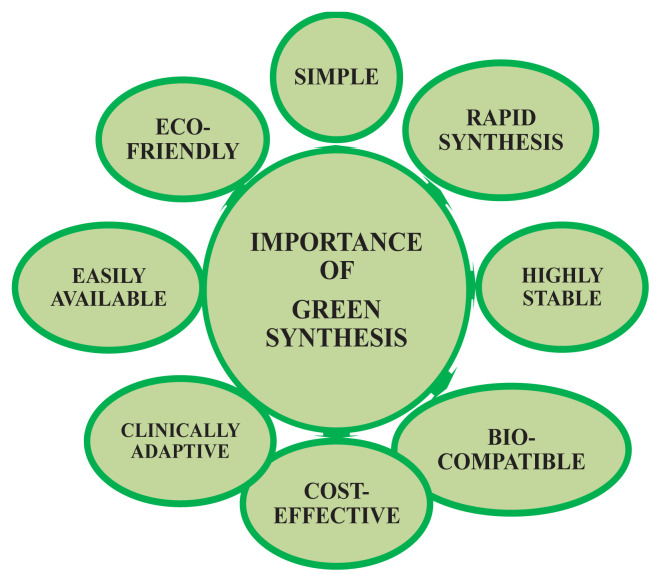
Importance of green synthesis.

Green synthesis methods for nanomaterials are needed as the existing methods are often toxic or entail costly physical and chemical processes. During chemical synthesis processes, toxic chemicals can be absorbed on various surfaces and exert adverse effects. Compared to chemical and physical methods, green synthesis methods do not need high temperatures or pressures, they are cost-effective and environmentally friendly, and they are easily scaled for large productions, as shown in Figure 3 [15]. Alongside these advantages, some limitations do also exist, such as the rate of reaction being comparatively low, the purity of samples being a challenge, and the impossibility of manipulating the material characteristics of natural products.

## 3. Green synthesis of metal and metal oxide nanomaterials

Green resources can act as reducing agents, capping agents, and oxidizing agents for the synthesis of metal and metal oxide nanomaterials. In the literature, different methods are available for the synthesis of nanomaterials, as summarized in Figure 1.

### 3.1. Green synthesis of nickel oxide (NiO) NPs

Nickel (Ni) is a transition metal that plays an important role in chemistry. In nano form, it reacts with oxygen and other metals to form nanocomposites, enhancing its properties. The synthesis of Ni NPs and NiO NPs using *Calotropis gigantea* extract was reported in the literature [12]. In that study, the extract acted as a reducing and capping agent. Both types of NPs were characterized through UV/Vis spectroscopy and the absorption peak was found at 400 nm for Ni NPs and at 415 nm for NiO NPs. Both types of NPs showed good antibacterial and catalytic activity. In green synthesis, a *Zea mays* silk extract was reported for the synthesis of NiO NPs, which were characterized by X-ray diffraction (XRD) and high-resolution transmission electron microscopy (HRTEM) and found to have diameters of 10–20 nm. These NPs were used in electrochemical energy storage devices [20].

**Figure 4:**
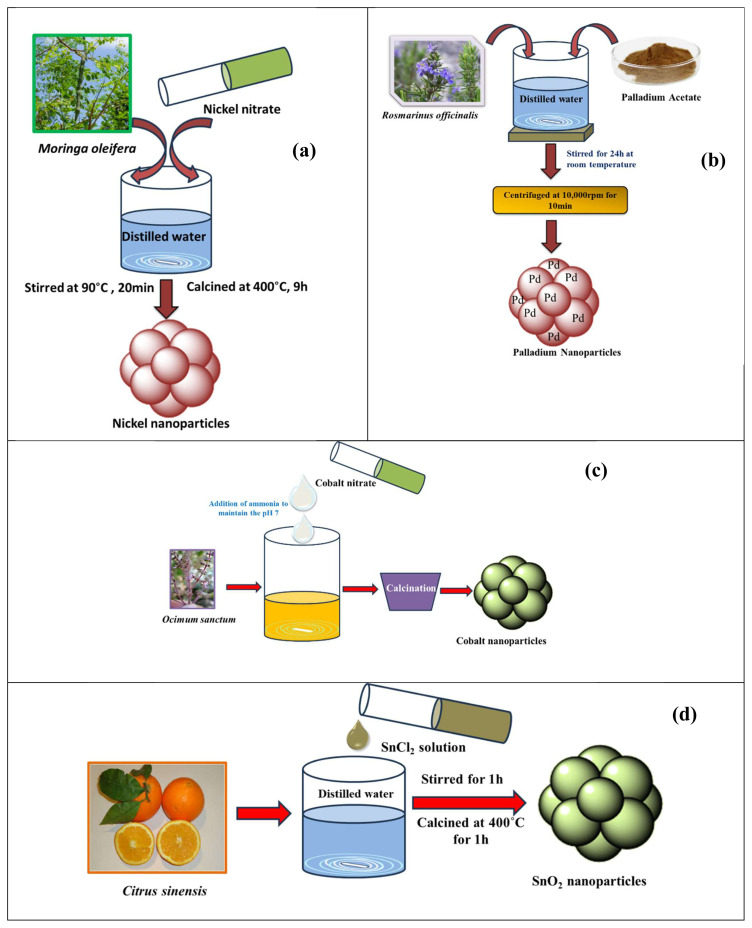
Synthesis of (a) NiO NPs [21]; (b) Pd NPs [37]; (c) Co NPs [50]; (d) Sn NPs [11].

NiO NPs synthesized using *Moringa oleifera* exhibited single crystalline face-centered cubic phases and had intense photoluminescence and antibacterial activities, as shown in Figure 4a [21]. Green NiO NPs synthesized using an extract of *Terminalia* plants exhibited emissions at 305.46 nm and 410 nm. They had improved cytotoxicity against breast cancer cells in a dose-dependent manner in the range of 0–100 μg/mL and were also used in biological and biomedical applications [22]. NiO NPs synthesized using a *Euphorbia heterophylla* plant extract could be used as an antimicrobial coating for biomedical and environmental applications. The UV-DRS spectra of the biosynthesized NiO NPs exhibited well-defined optical properties with an optical band gap of 3.24 eV and cytotoxicity against a human lung cancer cell line (A549) and human hepatocarcinoma (HepG2) [23]. NiO NPs prepared with fresh egg whites were subjected to MTT cytotoxicity testing against a human primary glioblastoma cell line (U87MG) commonly used in brain cancer research [24]. It was found that 50% of the cells died after exposure to the NPs at a concentration of 15.62 μg/mL. These NPs also exerted photocatalytic activity to remove methylene blue (MB) dye under UV light irradiation, with 79% degradation observed after 4 h.

Ni complex-functionalized Fe_3_O_4_ was utilized as a green and reusable catalyst for one-pot synthesis of polyhydroquinoline derivatives using pistachio leaf extract. It showed high conversion amounts and easy reusability with no loss of catalytic activity [25]. NiO NPs synthesized using *Aegle marmelos* leaf showed intense emission peaks at 363 and 412 nm and a band gap value of 3.5 eV. These NPs exerted good antibacterial activity and photocatalytic activity against 4-chlorophenol [26]. The synthesis of green NiO NPs using *Monsonia burkeana* leaves yielded good anticancer activity against A549 lung cancer cells. The average particle size of these NPs was found by HRTEM and XRD to be 20 nm [27]. NiO was synthesized using *Agathosma betulina* leaf extract for use in a p-type semiconductor and band gap values in the range of 3.6 to 4 eV were found [28]. Other similar studies on the synthesis of NiO and Ni NPs are given in Table 1 and the references [29–36].

### 3.2. Green synthesis of palladium (Pd) NPs

The synthesis of Pd NPs from *Rosmarinus officinalis* yielded good catalytic and biological properties, as shown in Figure 4b. The catalytic activity of the metal was investigated by Mizoroki–Heck reaction and the biological activity of the synthesized NPs was evaluated in terms of antibacterial and antifungal assessments against *Staphylococcus aureus*, *Staphylococcus epidermidis*, *Escherichia coli*, and *Micrococcus luteus* bacteria and *Candida parapsilosis*, *Candida albicans*, *Candida glabrata*, and *Candida krusei* yeast [37].

Pd NPs synthesized with *Solanum nigrum* showed spherical shapes with a size of 21.55 nm, and the presence of antioxidants and polyphenols in the extract was responsible for reduction and stabilizing properties [38]. Pd NPs synthesized with *Spirulina platensis* also had spherical shapes and a size range of 10–20 nm [39]. The highest absorption efficiency of the Pd NPs was obtained at pH 6 with contact time of 60 min, absorbent dose of 0.5 g/L, and lead concentration of 10 mg/L. Upon increasing the lead concentration from 10 to 150 mg/L, the removal percentage decreased from 87% to 32%, and when the absorbent dose was increased from 0.02 to 0.5 g/L, the removal percentage increased from 12% to 90%. Pd/Fe_3_O_4_ nanocomposites prepared from *Hibiscus tiliaceus* were used as a catalyst for the reduction of Cr(VI), 4-nitrophenol, and 2,4-dinitrophenylhydrazine. The flavonoids present in the extract acted as both reducing and capping/stabilizing agents [40]. A Pd/GO nanocomposite was also synthesized using *Thymbra spicata* and its recyclability and catalytic activity were studied [41]. Green Pd NPs synthesized using a lentinan extract had uniform distribution of graphene with high absorption ability. Efficient electron transfer was reported from graphene to the Pd NPs, which made the synthesized Pd NPs/FGO nanohybrid an effective nanocatalyst to be further utilized for 4-nitrophenol reduction. Recycling of the catalyst and good antimicrobial activity were also observed [42]. The green synthesis of Pd NPs/RGO using *Hippophae rhamnoides* was reported for the catalytic reduction of nitro aromatic compounds by NaBH_4_ [43]. Pd NPs synthesized using *Anogeissus latifolia* were found to be spherical in shape with average particle size of 4.8 ± 1.6 nm. The produced Pd NPs showed good catalytic activity and superior antioxidant properties at much lower NP doses [44]. The synthesis of Pd NPs was performed using *Ananas comosus* leaf extract as a reducing and stabilizing agent. These NPs were investigated for photocatalytic degradation of low-density polyethene and were concluded to be a useful material for the polymer industry [45]. Other similar studies are summarized in Table 1 and the references [46].

### 3.3. Green synthesis of cobalt (Co) NPs

The proteic sol-gel green method has been used to synthesize cobalt tungstate powder through agar-agar obtained from red seaweed, utilized further for battery electrodes. The long-term stability of the electrodes was confirmed by capacity retention of about 98% over 1000 charge–discharge cycles at a specific current of 1 A g^–1^ [47]. The green synthesis of cobalt oxide NPs was performed using *Sageretia thea* leaf extract as a chelating agent. These synthesized NPs were used for their antibacterial activity [48]. Cobalt ferrite NPs were successfully synthesized using the fungus *Monascus purpureus*. The production method was reported to be eco-friendly and easy. Transmission electron microscopy (TEM) analysis of these NPs confirmed their spherical shape with an average size of 6.5 nm. The NPs showed good antibacterial and antioxidant properties against all tested microbial pathogens and plants [49]. Cobalt ferrite and Ag-doped cobalt ferrite were synthesized using Tulsi seed (*Ocimum sanctum*) extract and garlic (*Allium sativum*) extract as shown in Figure 4c [50]. These materials were applied against gram-positive and gram-negative bacterial strains.

The XRD pattern of cobalt oxide powder produced with tamarind fruit extract by calcination of a polynuclear complex precursor at 1000 °C/2 h indicated the formation of the CoAl_2_O_3_ cubic spinel with a single phase. These NPs were used for their antimicrobial activity [51]. Cobalt ferrite synthesized with tomato (*Solanum lycopersicum*) leaf extract is widely used in medical applications. The crystalline size as determined by XRD was 17 ± 1 nm [52]. Fluorescent metallic oxide NPs have been synthesized with *Aspergillus nidulans* at room temperature. Fourier transform infrared (FTIR) analysis showed that protein capping stabilized the NPs [53]. The single phase of a CoFe_2_O_4_ sample was obtained with the wet ferritization method using an aqueous extract of *Sesamum indicum* seed. The XRD pattern of the sample calcined at 800 °C/1 h indicated the formation of the CoFe_2_O_4_ spinel type with good crystallinity. The tested cobalt ferrite was not cytotoxic at the examined concentrations against different microbial strains, but it demonstrated potential for use in in vitro applications [54]. Other synthesized cobalt oxide NPs are presented in Table 1 and the references [55,56].

### 3.4. Green synthesis of tin oxide (SnO_2_) NPs

Tin dioxide NPs were synthesized in different concentrations using an extract from the peel of *Citrus sinensis* as a reducing agent, as shown in Figure 4d. Tin oxide achieved better activity because of its stability, in addition to being nontoxic, low-cost, and easily available. It was able to degrade a very high percentage of MB and could be used in air and water purification techniques [11].

Green SnO_2_ NPs synthesized using *Lycopersicon esculentum* peel extract were further studied for photocatalytic activity in the degradation of MB, achieving a rate of 100% within 120 min. A better degradation rate was obtained compared to both NPs synthesized with other synthetic routes and commercially available bulk SnO_2_ [17]. The green synthesis of SnO_2_ NPs using jujube fruit was performed to obtain a systematic photocatalyst for use in the decay of two hazardous organic dyes, namely MB and Eriochrome Black T, under sunlight. Degradation efficiency of 90% and 83% was respectively obtained [16]. The synthesis of SnO_2_ NPs using *Psidium guajava* was also reported, with sizes ranging between 8 and 10 nm. These NPs were highly effective in degrading RY186 dye. The antimicrobial activity of these NPs was also evaluated against *S. aureus*, *Bacillus subtilis*, and *E. coli*. Levels of antimicrobial activity were higher against *S. aureus* compared to *B. subtilis* and *E. coli*. The antioxidant activity of SnO_2_ NPs on vitamin C was also studied using the DPPH assay [57]. A ZnSnO_3_ nanocluster synthesized using *Aspalathus linearis* plant extract was annealed at 500 °C. The morphology of the product was investigated and the average size of the NPs was found to be 16.5 to 20.5 nm. UV-Vis spectra showed an absorption edge at 354 nm and a band gap of 3.50 eV, which confirmed that the tin NPs had good optical properties [58]. Other studies on different green sources used for tin oxide NPs are available in the literature [59,60].

### 3.5. Green synthesis of iron (Fe) NPs

**Figure 5:**
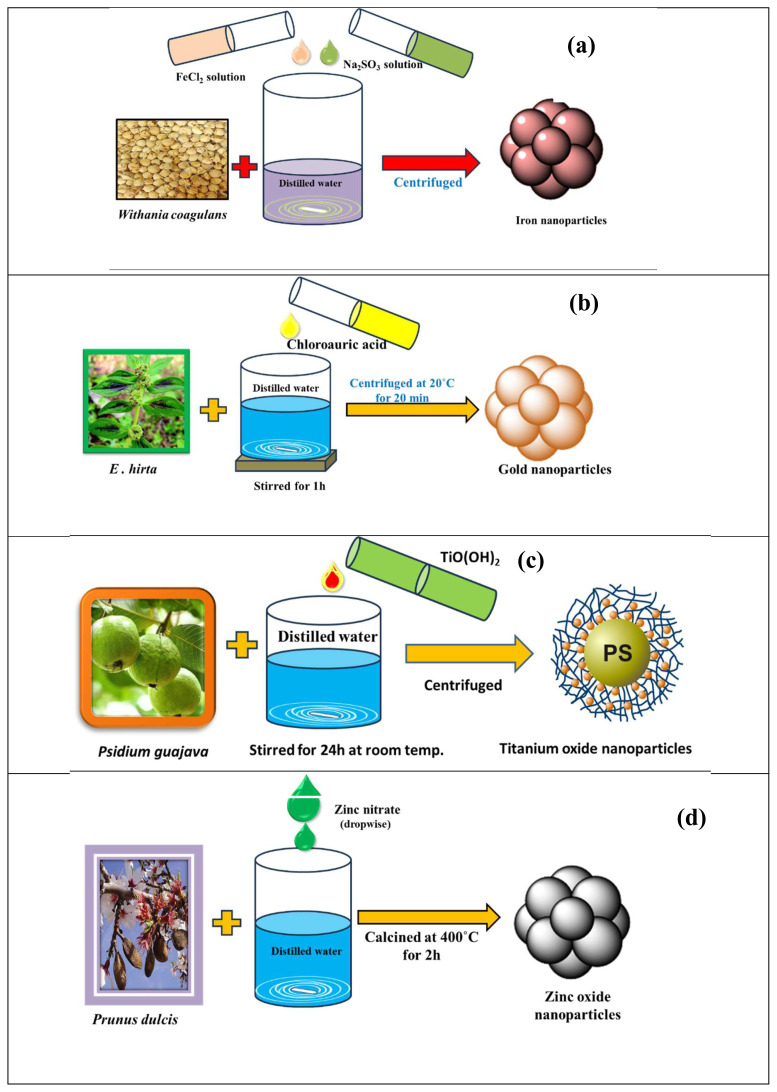
Synthesis of (a) iron NPs [66]; (b) gold NPs [70]; (c) titanium NPs [76]; (d) zinc NPs [77].

The principles of green chemistry, waste prevention, energy efficiency, safer solvents, and benign precursor materials have become fundamental considerations in synthesizing NPs [61]. The green synthesis of iron NPs using *Azadirachta indica* leaf was performed in a study that evaluated the effect of size and the concentration of polyphenols, and the efficiency against petroleum refinery waste water with high COD values and nitrates was determined. The size of the Fe NPs was found to be in the range of 98–500 nm. It was evident that the polyphenol content together with the Fe NPs increased the production of reactive oxygen species (ROS). The accumulation of these NPs in cytoplasm occurred due to size variations; increased antibacterial activity was also observed. The zones of inhibition were 25 nm for *E. coli*, 29 nm for *Pseudomonas aeruginosa*, and 30 nm for *S. aureus* [62]. Similarly, Fe NPs produced with *Aspergillus niger* were utilized for Cr(VI) removal from aqueous solution, showing >99% removal of Cr at 40 °C and pH 3 with an adsorbent dose of 2.5 g/L. Fe NPs were regenerated using NaOH solution and retained 79.7% of their metal removal capacity for five successive cycles of absorption and desorption [63]. The green synthesis of two types of FeO NPs using *Cucurbita moschata* leaves and *Beta vulgaris* stalks was also reported [64]. These materials were further used for the adsorption of two dyes, namely Bordeaux red and tartrazine. Both materials showed different adsorption capacities varying from 59 to 64 mg g^–1^. An extract of yerba mate was prepared for iron NPs, which were applied for the removal of Cr(VI) from aqueous solution at pH 3 with two concentration ratios of Cr(VI) and iron NPs, i.e., 1:3 and 1:05 [65]. The rate of the reaction was compared to that of a commercial nanoscale zerovalent iron solution. The rapid rate of the NPs allowed the removal of pollutants in soil and ground water. An extract of *Withania coagulans* was used for the synthesis of iron oxide NPs, as shown in Figure 5a, and the NPs were applied for antimicrobial activity and photocatalytic degradation [66].

Zerovalent Fe NPs synthesized using mango peel had a structure similar to that of Fe^+2^/Fe^+3^ complex islands over metallic iron [67]. The role of different polyphenol compounds in stabilizing the NPs and changes in surface characteristics and stability against desorption and biodegradation were described. Fe NPs were also synthesized with an aqueous extract of two plants, namely *Terminalia bellirica* and *Moringa oleifera* [68]. Total phenolic contents were highest for the *Terminalia* extract (3581.36 ± 2.38 μg/mL). These NPs were used for antibacterial activity. Antioxidant activity was also higher with *Terminalia* compared to the *Moringa oleifera* extract. In another study, *Avicennia marina* flowers were used to control the size of iron NPs and sizes of about 100 nm were reported [69]. These promising greener materials may have important roles in applications requiring antitoxicity or dye degradation. They are compatible with electrical materials, applications in the electronics industry, and the design of high-quality materials.

### 3.6. Green synthesis of gold (Au) NPs

Various NP reduction reagents are available, such as NaBH_4,_ LiBH_4_, cetyltrimethylammonium bromide (CTAB), and NaOH. They have functions of surface modification with suitable capping ligands to prevent the self-aggregation of Au NPs. To control aggregation, various plant extracts are also utilized. Au NPs were produced using a leaf extract of *Euphorbia hirta*, as shown in Figure 5b, and they were found to be environmentally friendly with antibacterial activities against *E. coli*, *P. aeruginosa*, and *Klebsiella pneumoniae* strains [70].

Au NPs synthesized using *Schisandra chinensis* fruit were assembled on polystyrene beads. After characterization, the Au NPs were used as a heterogeneous catalyst to promote a one-pot sequential reaction for the synthesis of bifunctionalized chromeno([2,3-*d*]pyrimidin-2-yl)phenol derivatives [71]. The UV-Vis spectra of Au NPs synthesized with *Sphaeranthus indicus* showed a surface plasmon resonance peak at 531 nm [72]. TEM revealed a spherical shape with mean particle size of 25 nm. These Au NPs were used for their antioxidant and photochemical activity. The cytotoxicity of Au NPs synthesized with *Olea europaea* and *Acacia nilotica* was evaluated by MTT assay against breast (MCF-7), colon (TCT-116), and hepatocellular (HCepG-2) cancer cell lines [73]. The size of the Au NPs was found to be less than 10 nm at the chosen concentration. Additionally, the combination of 0.3 mL of *Simarouba glauca* leaf extract and 2.7 mL of gold solution was shown to provide superior results in terms of antimicrobial activity [74].

### 3.7. Green synthesis of titanium oxide (TiO_2_) NPs

TiO_2_ NPs were synthesized using the aqueous leaf extract of *Aloe barbadensis*, which acted as a reducing and fabricating agent. Due to their unique properties, the NPs could be widely used as antioxidant agents. TiO_2_ NPs synthesized with *Sesbania grandiflora* showed 100% peak intensity with a z-average value of 620 nm by dynamic light scattering (DLS) analysis. TEM analysis confirmed that the NPs were 20–40 nm in size. XRD and energy dispersive X-ray (EDX) analysis confirmed the crystalline rutile structure of the TiO_2_ NPs [75]. TiO_2_ NPs synthesized with *Psidium guajava* were analyzed by field emission scanning electron microscopy (FESEM) and were found to have spherical shape and sizes in the range of 32.58–35.25 nm. The synthesis route for these NPs is given in Figure 5c. They were used for in vitro cytotoxicity. The phenolic contents of the leaf extract and the NPs were respectively found to be 85.4 and 18.3 mg TA/g [76].

### 3.8. Green synthesis of zinc oxide (ZnO) NPs

ZnO NPs were prepared using *Prunus dulcis* (almond gum). The synthesis route of these NPs is provided in Figure 5d. The extract showed effective antibacterial activity against *S. aureus* and *E. coli*. The UV analysis spectrum showed an absorption peak at 243 nm and a band gap value of 5.17 eV. XRD analysis confirmed a wurtzite structure with average crystalline size of approximately 30 nm [77].

The UV-Vis spectra of ZnO NPs prepared with *Camellia japonica* leaf extract showed an absorption peak at 301 nm. The crystalline ZnO NPs were 20 nm in size. The synthesized NPs were used in a biological system as optical sensors [78]. Small crystalline size was achieved with increased surface area, leading to good antibacterial activity. The synthesis of ZnO NPs using a hydrothermal method and plant extract of *Justicia adhatoda* was also studied [79]. The average crystalline size was found to be 36 nm and the band gap was 3.36 eV, which helped enhance the anticancer and antibacterial activities of the NPs. The selected area electron diffraction (SAED) pattern showed highly crystalline morphology. ZnO NPs synthesized with *Hydnocarpus alpinus* had spherical morphology with diameters of 38.84 nm and high phase-purity [80]. ZnO NPs showed scavenging activity against 2,2-diphenyl-1-picrylhydrazyl (DPPH) free radicals. At a basic pH, photocatalytic activity was observed for MB degradation at a rate of about 96%. Green ZnO NPs synthesized with *Aristolochia indica* exhibited strong bactericidal properties against *E. coli* and the average size was 22.5 nm with zeta potential of –21.9 ± 1 mV [81]. ZnO NPs synthesized using an extract of *Selaginella convoluta* were characterized by their FTIR spectra, which indicated that polyphenols acted as capping ligands. These synthesized NPs were used in biomedical applications for pain management [82].

ZnO NPs synthesized using *Euphorbia heterophylla* leaf extract were characterized with a hexagonal wurtzite structure [83]. The optical energy band gap value was found to be about 3.15 eV by DRS. TEM analysis revealed an average size of 40 nm. The ZnO NPs showed good antibacterial and anticancer activities and they were evaluated against lung (A549) and hepatocarcinoma (HepG2) cancer cell lines. Antibacterial and antifungal activities were evaluated by well diffusion method based on minimum inhibitory concentrations. The maximum zones of inhibition of ZnO NPs synthesized using an extract of *Aeromonas hydrophila* (25 μg/mL) were reported for *Pseudomonas aeruginosa* (22 ± 1.8 mm) and *Aspergillus flavus* (19 ± 1.0 mm). The ZnO NPs were characterized by atomic force microscopy and a size of 57.72 nm was reported together with the topological appearance in 3D profile on the surface on the nanoscale [84].

ZnO NPs synthesized using *Mirabilis jalapa* were evaluated for the presence of phenolic- and flavonoid-like properties due to the presence of different functional groups on the particle surface. By XRD characterization of the NPs, the crystalline size was found to be 12.9 nm. Bimetallic ZnO/Ag NPs exhibited antibacterial (zones of inhibition of up to 25 mm) and antileishmanial properties [85]. ZnO NPs synthesized with *Trianthema portulacastrum* leaf extract were evaluated for antioxidant activity against DPPH. Photocatalytic activity was also evaluated for Synozol Navy Blue (KBF) textile dye and a degradation rate of 91% was reported after 159 min [86]. ZnO NPs synthesized using *Trifolium pratense* flower extract were tested against standard strains of *S. aureus*, *P. aeruginosa*, and *E. coli* and the best results were obtained for *E. coli*. XRD characterization of the NPs showed crystalline size of 60–70 nm and total reflection X-ray fluorescence (TXRF) showed an intense signal at 8.63 KeV for analysis performed at 50 kV and 600 μA [87].

ZnO NPs synthesized with *Punica granatum* peel extract exhibited cytotoxicity against both normal human colon cells and cancerous cells. They exhibited cell-death activities for both types of cells at a concentration of ≥31.25 μg/mL. TEM analysis of the NPs revealed a hexagonal shape and averages size of 32.98 nm at 600 °C and 81.84 nm at 700 °C. They also displayed good antibacterial activity [88]. ZnO NPs synthesized using *Medicago sativa* were used in antimicrobial testing against bacterial strains of *Staphylococcus epidermidis* (ATCC49461), *Saccharomyces cerevisiae* (MG012794), and *Lactobacillus* (ATCC334) and yeast (*Candida albicans* ATCC10231). After nano-ZnO treatment, the fluorescence indicated the formation of vacuolization and the deformation of yeast cells. EDX analysis of these NPs with signals of 1 keV and 8.5 keV highlighted the presence and chemical distribution of ZnO NPs while TEM analysis showed an average size of 10 nm [89]. Synthesis of ZnO NPs using *Cucurbita pepo* leaf extract was performed to induce cytotoxicity against the proliferation of MG63 osteoblast-like cells and reduction in cell proliferation was confirmed. TEM analysis showed a spherical shape and average particle size of 8 nm [90].

ZnO NPs synthesized using *Costus woodsonii* leaf extract showed crystalline hexagonal wurtzite structures and an optical band gap value of 3.18 eV. The ZnO NPs were also prepared by boiling the leaf extract to narrow the band gap and values of approximately 2.68–2.77 eV were reported [91]. *Raphanus sativus* root extract was used to synthesize ZnO NPs and their antimicrobial activity was studied against *E. coli* [92]. ZnO NPs were synthesized using *Garcinia xanthochymus* for the photodegradation of MB in the presence of UV rays and sunlight. They exhibited antioxidant activity against the DPPH free radical. SEM analysis of these NPs showed spongy cave-like structures and the photoluminescence spectra showed four emission edges at 397, 436, 556, and 651 nm [93]. The antioxidant activity of ZnO NPs produced using *Tecoma castanifolia* was found to increase with concentration, leading to increased radical scavenging activity. An IC_50_ value of 65 μg/mL was obtained as a measure of anticancer activity, revealing the good cytotoxic effects of ZnO NPs against the proliferation of A549 cells [94].

ZnO NPs produced using *Pongamia pinnata* extract were crystalline in structure at 350 °C. They showed antibacterial activity against pathogenic bacteria and successfully minimized infection [95]. ZnO NPs were synthesized using *Ruta chalepensis* leaf extract and by a chemical method, and the properties of the two types of samples were compared. It was found that the green ZnO NPs had an average size of 17.72 nm and band gap value of 2.86 eV, which were lower than the values obtained for the chemically synthesized NPs. The green ZnO NPs also achieved better degradation of Malachite green (MG) compared to the chemically produced ZnO NPs [96]. ZnO NPs synthesized using *Coriandrum sativum* leaf extract calcined at 100 °C and 550 °C had band gaps of 3.56 and 3.72 eV, respectively, and crystalline sizes of 60.85 and 55.13 nm, respectively. However, the ZnO NPs produced at 550 °C had better structural properties compared to those produced at 100 °C [97]. ZnO NPs were synthesized using *Thymus vulgaris* leaf extract by hydrothermal method. An in vitro DPPH assay to evaluate antioxidant activity showed prominent activity (<75%) at higher concentrations [98]. XRD analysis revealed average NP crystalline sizes of 46.74, 132.54, and 779.38 nm for 1, 0.5, and 0 mL of *Thymus* leaf extract, respectively. Table 2 presents studies on the synthesis of iron, gold, TiO_2_, and ZnO NPs and their applications.

### 3.9. Green synthesis of silver (Ag) NPs

**Figure 6:**
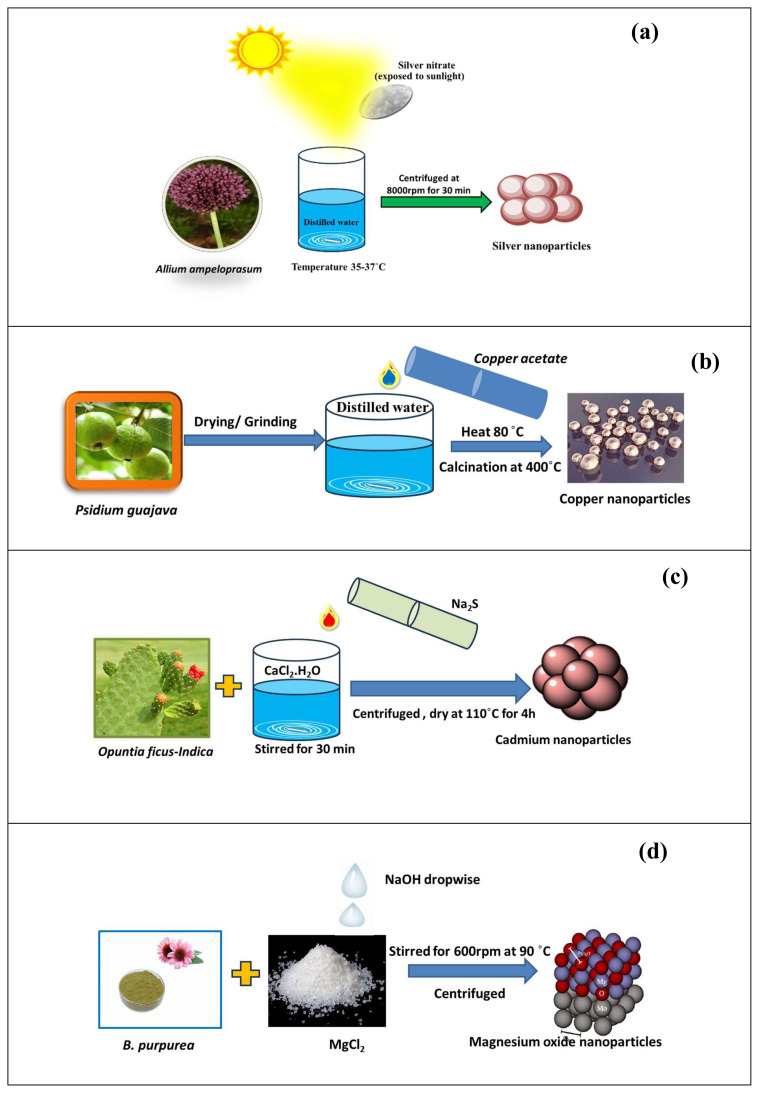
Synthesis of (a) silver NPs [99]; (b) copper NPs [136]; (c) cadmium NPs [127]; (d) magnesium NPs [131].

Ag NPs synthesized using *Allium ampeloprasum* had high levels of activity against the HeLa cell line with an IC_50_ value of <25 μg/mL. Total phenolic contents were 15.58 μg/mL and 10.94 μg/mL for the extract and the NPs at a concentration of 150 μg/mL, respectively [99]. The synthesis route of these NPs is shown in Figure 6a.

Ag NPs synthesized with *Crataegus pentagyna* were used in catalysis for the degradation of the organic dyes Rhodamine B (RhB), eosin (EY), and MB with rates of 85%, 70%, and 78% achieved respectively under sunlight. Antibacterial activity against seven ATCC strains of bacteria and eight strains of drug-resistant bacteria was also reported [100].

The in vitro cytotoxicity of NPs against the MCF-7 and AGS cell lines was assayed using Ag NPs prepared with different concentrations of *Crataegus microphylla*. Doxorubicin was used as a positive control. Excellent inhibition of the growth of the MCF-7 and AGS cell lines was reported [101]. NPs produced with *Annona muricata* extract showed antiproliferative effects against A549 with elevated activity in nano form. The anticancer activity of the NPs was studied in the context of the upregulation and downregulation of apoptotic (Bax and caspase) and antiapoptotic (Bcl-2) genes along with their functional groups [102]. The synthesis of Ag NPs using *Handroanthus heptaphyllus* yielded a maximum absorption peak close to 440 nm, indicating that the nanostructure had a hydrodynamic diameter of 10 nm for Ag NPs [103].

Ag NPs synthesized using *Tamarindus indica* fruit extract were used for anticancer activity. Evaluation of the cytotoxicity of these NPs showed dose-related effects against breast cancer cells (MCF-7); using the MTT assay, the IC_50_ value was found to be 20 μg/mL [104]. The synthesis of Ag NPs using *Andrographis paniculata* was performed to induce levels of ROS, reduce the activity of thioredoxin reductase, and thus shift the redox homeostasis of the particles [105]. After the synthesis of Ag NPs using reishi mushrooms, the highest antioxidant activity in the form of DPPH scavenging was found to be 76.45% at 250 mg/L. The analysis of DNA cleavage activity indicated that the Ag NPs caused single-strand DNA cleavage for 30 and 60 min at 50 and 100 mg/L concentrations [106]. Ag NPs synthesized using *Rheum ribes* were evaluated for cytotoxicity against the MDA-MB-231 breast carcinoma cell line. The IC_50_ values of the NPs ranged from 165 to 99 μg/mL against the MDA-MB-231 cell line for 24 h and 48 h of exposure [107].

The synthesis of Ag NPs using *Fumaria parviflora* was performed and an absorption peak at 460 nm was observed. The MMT assay revealed the prevention of viability in human breast cancer cells [108]. The MMT assay was also applied for Ag NPs synthesized with *Delonix regia*, exhibiting better antiproliferative activity against the A549 cell line in comparison to the SiHa cell line. The circular dichroism was performed to study the decrease in alpha-helical content in the perturbation of the secondary structure [109]. Ag NPs synthesized using *Calotropis gigantea* leaf extract were studied for larvicidal properties and antimicrobial activity against gram-negative and gram-positive bacteria [110]. Ag NPs were also obtained using the water extract of marine algae (*Gracilaria dura*). They exhibited powerful antimicrobial, anticoagulant, and anticancer activities [111]. A broth microdilution test showed that Ag NPs prepared using red algae (*Portieria hornemannii*) had high levels of antimicrobial activity with very low MIC values (0.51 μg/mL for *Candida albicans*, 0.26 μg/mL for *E. coli*). In a study of prebiofilm effects, an 81% reducing effect on biofilm formation was achieved at 0.51 μg/mL. The highest reduction rate in postbiofilm studies was 73.5%, achieved with 2.04 μg/mL Ag NPs [112]. An aqueous extract of green algae (*Botryococcus braunii*) had the potential to stabilize Ag NPs. The NPs were found to be efficient for the reduction of 2-nitroaniline and in the synthesis of 2-arylbenzimidazoles [113].

Ag NPs synthesized with *Berberis vulgaris* extract had spherical shapes and sizes of 30–70 nm [114]. Jackfruit was used for the synthesis of Ag NPs, and its seeds contain jacalin. Jacalin recognizes and binds to the O-glycoprotein of tumor-associated T-antigenic disaccharide. Thanks to the strong interaction of these NPs with cancer cells, they could be used in cancer therapy. The particle size was found to be 22.53 ± 1.51 nm by HR-TEM [115]. Ag NPs synthesized with *Combretum erythrophyllum* plant leaves were characterized and the particle size was found to be 13.62 nm. The synthesized NPs were used for their antibacterial activity against gram-positive and gram-negative bacteria [116]. Ag NPs synthesized with *Allium cepa* showed high levels of antidiabetic activity by inhibiting carbohydrate metabolites such as α-amylase and α-glycosidase. They exhibited good antioxidant activity by scavenging free radicals [117]. Ag NPs synthesized with *Laminaria japonica* by hydrothermal process were successfully evaluated for their metallic, optical, and structural properties in a steam autoclave at 100 kPa and with a 20-min time duration [118].

The synthesis of Ag NPs using *Ampelocissus latifolia* was confirmed by color changes in UV analysis with an absorption peak at 436 nm [119]. Ag NPs synthesized with *Rhododendron ponticum* were studied for their anticarcinogenic properties. The MTT test was performed using the MCF-7 and 4T1 cell lines in cell culture. Antibacterial and antibiofilm inhibition was achieved against pathogens such as *Enterococcus durans* [120]. Ag NPs synthesized using *Nauclea latifolia* fruit extract were found to exert antimicrobial and antifungal activity against *Pseudomonas aeruginosa*, *E. coli*, and *Aspergillus niger* with high sensitivity. This aqueous extract had a broad spectrum of activity compared to a menthol extract [121]. Ag NPs synthesized using *Annona reticulata* were exposed to fourth-instar larvae at different concentrations (3–20 μg/mL) for 24 h and maximum mortality was obtained at a final concentration of 1 mg/mL. The LC_50_ lethal concentration value was 4.43 μg/mL and the LC_90_ value was 13.96 μg/mL [122]. The formation of Ag NPs using *Madhuca longifolia* was observed at 40 °C after 20 min and a significant UV spectra peak was found at 436 nm [123].

### 3.10. Green synthesis of copper oxide (CuO) NPs

CuO NPs synthesized using *Tinospora crispa* had benefits including being harmless and low-cost with a simple preparation method. The typical absorption peak of the CuO NPs occurred at 383 nm and the band gap energy value was 1.32 eV [124]. The synthesis and characterization of CuO-ZnO nanocomposites prepared using *Thymus vulgaris* was also performed and sizes of 10–20 nm were reported. The flavonoid and phenolic constituents were confirmed by FTIR. The phenolic constituents drove the reduction of the CuCl_2_ and functioned as capping ligands on the surfaces of the CuO NPs. These NPs exhibited good catalytic activity. The catalyst was retrieved and reused many times and there was no decrease in catalytic activity [125]. Biogenic CuO NPs synthesized using *Psidium guajava* had an optical band gap value of 2.5 eV as shown in Figure 6b. The effectiveness of the NPs was tested based on the degradation of industrial dyes (e.g., NB and RY160 relative to MB and Congo red). CuO NPs synthesized with *Melissa officinalis* extract were stable and served as an efficient catalyst with antibacterial activity [126].

### 3.11. Green synthesis of cadmium (Cd) NPs

CdS NPs produced by green synthesis are eco-friendly and naturally renewable. The fruit of *Opuntia ficus-indica* acted as a stabilizing and capping agent in the production of highly homogeneous CdS spherical NPs with particle sizes in the range of 3–5 nm. The synthetic process used for these NPs is given in Figure 6c. *Opuntia ficus-indica* fruit sap was also used in the synthesis of CdO semiconductor quantum dots. CdS was determined by UV-Vis analysis at 323 nm and DLS analysis yielded a d_50_ value of 9.56 nm. The main applications of this synthesized green material are in solar cells [127].

The synthesis of CdO NPs was performed using turmeric extract act as a reducing agent. The optical band gap value of the CdO NPs was found to be 5.8 eV. The antibacterial behavior of the green synthesized NPs was tested against *Pseudomonas aeruginosa*, *Klebsiella pneumoniae*, *Staphylococcus aureus*, and *Escherichia coli* using agar well diffusion. Anticancer activity was also determined against human colon cancer cells (HT29) using the MTT assay [128]. CdO NPs synthesized using olive were utilized for antifungal activity. XRD analysis of the CdO NPs revealed an average crystallite size of 20 nm. The particle size was estimated as 32 nm using a particle size analyzer. The weight loss as measured by a TG-DTA curve was 0.98% [129].

### 3.12. Green synthesis of magnesium oxide (MgO) NPs

Mg NPs synthesized using *Penicillium chrysogenum* were evaluated under the influence of different gamma doses. The antimicrobial activity of the Mg NPs was examined against common pathogenic bacteria, unicellular fungi, and multidrug-resistant pathogens [130]. Antibacterial activity was also reported for MgO NPs, which was synthesized using *Bauhinia purpurea* leaf extract. These NPs were further utilized with antibacterial activity against *Staphylococcus aureus* and exhibited good activity [131].

MgO NPs synthesized by coprecipitation route at room temperature using brown marine algae (*Turbinaria ornata*) with antimycobacterial activity were applied against *M. tuberculosis* H39Rv and a luciferase reporter phage assay revealed 73% relative light unit reduction [132]. MgO NPs synthesized using marine algae (*Sargassum wightii*) were highly stable at 19.8 mV and the particle size was 68.06 nm. These NPs were used for their antifungal, antibacterial, and photocatalytic activities [133]. *Withania somnifera* has been used in Ayurvedic medicine in India. MgO NPs synthesized with *W. somnifera* extract were used for electrochemical sensing and antifungal and cytotoxic activity [134]. NPs synthesized with *Pisonia alba* showed good antioxidant activity. They exhibited strong fungicidal activity against *A. flavus and F. solani*. Good antioxidant properties were also exhibited with *P. alba* leaf extract in DPPH and FRAP assays [135]. A summary of the synthesis and application of these NPs is provided in Table 3.

## 4. Toxicity and safety of green nanomaterials

Together with their smaller sizes and unique properties, nanomaterials and nanocomposites affect the environment and human health adversely with long exposure or high quantities. Various consumer products and industries use nanomaterials as reactants or intermediates. Long-term exposure to these particles affects organs of the body such as the liver, spleen, and skin. If the concentration of NPs increases in the blood, they are then circulated throughout the body, potentially causing fatal organ damage. According to the literature, silver is deposited in the liver and spleen while gold is deposited in the liver [137,138]. Similarly, exposure to nanomaterials adversely affects the environment and especially aqueous reservoirs. These particles can enter the food chain via aqueous media and negatively affect the soil characteristics and organisms living in the soil such as worms and microorganisms [139].

Nanomaterials enter human and animal bodies through inhalation and ingestion; they are also absorbed by endocytosis. After entering the body, they generate ROS that damage different systems via mitochondrial breakdown, mitochondrial dysfunction, DNA damage, and protein denaturation. This leads to cytotoxicity and genotoxicity [140,141]. Limited data are available on assessments of the toxicity of green nanomaterials, although many researchers are currently working on this topic.

It is essential to assess the risks associated with nanomaterials, addressing uncertainties in manufacturing and usage processes. Proactive strategies must be developed for risk management, including preventive measures for toxic exposure. It is necessary to validate processes and execute precautions regularly, conduct exposure-based inspections and maintain vigilance, implement preemptive safety protocols to prevent accidents, initiate health assessments, and provide comprehensive training and information for individuals handling toxic materials.

## 5. Challenges of green synthesis

The advancement of green nanotechnology faces several challenges, including technical obstacles, the toxicity of NPs, adherence to regulatory policies governing their synthesis, and the industrial scaling-up of procedures. These factors impede the growth of the field. For green synthesis, the standard quality of raw materials is an important parameter for the consistency of the produced NPs. Therefore, when selecting raw materials, cost-effectiveness and economic feasibility are important criteria along with practicability [142]. Reproducibility is a major concern when using green nanomaterials [143].

## 6. Conclusions

The exploration of environmentally friendly methods for synthesizing metal and metal oxide NPs has been an important focus of research for many years. Various natural sources, including plant extracts, bacteria, fungi, and yeast, have been utilized with this aim. Notably, plant extracts have demonstrated significant effectiveness as both stabilizing and reducing agents. Different plant components such as stems, leaves, fruits, and seeds can be employed in this process. The rich presence of polyphenols in these natural extracts plays a crucial role in facilitating reduction, capping, and stabilization. The type and quantity of the polyphenols have direct impacts on the resulting particle size. Green NPs may be further utilized in various applications in the photocatalytic, electronic, and biomedical fields. Researchers increasingly favor green synthesized NPs with better catalytic activity.

## 7. Future perspectives

To foster sustainable and secure nanotechnology in the future, there is a need for clear design guidelines in production, swift toxicology analysis and clear protocols for assessing the safety of MPs, and increased demand from end markets to ensure broader applications and commercialization. It is necessary to gain a deeper understanding of the underlying reaction mechanisms in green approaches, employ improved characterization techniques, and enhance data analysis. These efforts will establish a strong foundation for eco-friendly and sustainable nanotechnology. While substantial advancements have been made in laboratory settings, the successful scaling-up of nanomaterial synthesis for real-world applications necessitates a thorough comprehension of the synthesis mechanisms and key components. In the future, research and development efforts should shift from laboratory-based work to the industrial-scale implementation of green materials and NP synthesis.

## Figures and Tables

**Table 1:** Synthesis of Ni, Pd, Co, and Sn NPs from biological sources and applications.

Name of material	Source	Nanoparticles	Applications	Reference
Citrus sinensis	Peel	Tin	Photocatalytic	[11]
Jujube	Fruit	Tin	Photocatalytic	[16]
Lycopersicon esculentum	Peel	Tin	Catalytic	[17]
Moringa oleifera	Leaves	Nickel	In vitro cytotoxicity	[21]
Terminalia chebula	Leaves	Nickel	Biomedical	[22]
Euphorbia heterophylla	Leaves	Nickel	Anticoagulant, cytotoxicity	[23]
Fresh egg	Albumen	Nickel	Cytotoxicity	[24]
Pistachio	Leaves	Nickel	Cytotoxicity	[25]
Aegle marmelos	Leaves	Nickel	Pharmaceutical	[26]
Monsonia burkeana	Leaves	Nickel	Anticancer activity	[27]
Agathosma betulina	Leaves	Nickel	-	[28]
Eichhornia crassipes	Leaves	Nickel	Hydrogen production	[29]
Neem	Leaves	Nickel	-	[30]
Calendula officinalis	Leaves	Nickel	Antiesophageal	[31]
Grapes	Seeds	Nickel	Biological activity	[32]
Limonia acidissima	Fruits	Nickel	Antioxidant activity	[33]
Plectranthus amboinicus	Leaves	Nickel	Antifungal activity	[34]
Cactus	Leaves	Nickel	Energy storage	[35]
Nigella sativa	Fruits	Nickel	Catalytic activity	[36]
Rosmarinus officinalis	Leaves	Palladium	Antibacterial	[37]
Solanum nigrum	Leaves	Palladium	Antimicrobial	[38]
Spirulina platensis	Leaves	Palladium	Antimicrobial	[39]
Hibiscus tiliaceus	Leaves	Palladium	Catalytic	[40]
Thymbra spicata	Leaves	Palladium	Catalytic	[41]
Lentinan	Plant	Palladium	Catalytic	[42]
Hippophae rhamnoides	Leaves	Palladium	Catalytic	[43]
Anogeissus latifolia	Leaves	Palladium	Catalytic	[44]
Chamomile	Flowers	Palladium	Catalytic activity	[46]
Red seaweed	Algae	Cobalt	Antimicrobial	[47]
Sageretia thea	Bacteria	Cobalt	Antimicrobial	[48]
Monascus purpureus	Fungus	Cobalt	Antioxidant	[49]
Allium sativum and Ocimum sanctum	Seed	Cobalt	Antibacterial	[50]
Tamarind	Fruits	Cobalt	Antimicrobial	[51]
Solanum lycopersicum	Plant	Cobalt	Medical	[52]
Aspergillus nidulans	Fungus	Cobalt	-	[53]
Sesamum indicum	Seed	Cobalt	Antimicrobial	[54]
Hibiscus rosa-sinensis	Leaves	Cobalt	Biomedical	[55]
C. sativum and A. sativum	Seeds and cloves	Cobalt	Photocatalytic	[56]
Psidium guajava	Leaves	Tin	Photocatalytic	[57]
Aspalathus linearis	Plant	Tin	-	[58]
Simarouba glauca	Leaves	Tin	Photocatalytic	[59]
Camellia sinensis	Leaves	Tin	Photocatalytic	[60]

**Table 2:** Synthesis of iron, gold, TiO_2_, and ZnO NPs from biological sources and applications.

Name of material	Source	Nanoparticles	Applications	Reference
*Azadirachta indica*	Leaves	Iron	Antibacterial	[62]
*Aspergillus niger*	Fungus	Iron	-	[63]
*Cucurbita moschata* and *Beta vulgaris*	Leaves and stalks	Iron	Photocatalytic	[64]
Yerba mate	Leaves	Iron	Removal of pollutant	[65]
*Withania coagulans*	Leaves	Iron	Antibacterial	[66]
Mango	Peel	Iron	-	[67]
*Terminalia bellirica* and *Moringa oleifera*	Leaves	Iron	Antibacterial	[68]
*Avicennia marina*	Flower	Iron	Dye degradation	[69]
*Euphorbia hirta*	Leaves	Gold	Antibacterial	[70]
*Olea europaea* and *Acacia nilotica*	Fruit	Gold	Cytotoxicity	[73]
*Simarouba glauca*	Leaves	Gold	Antimicrobial	[74]
*Sesbania grandiflora*	Embryo	TiO_2_	Antibacterial	[75]
*Psidium guajava*	Leaves	TiO_2_	In vitro cytotoxicity	[76]
*Prunus dulcis*	Almond	ZnO	Antimicrobial	[77]
*Camellia japonica*	Leaves	ZnO	Biological sensor	[78]
*Justicia adhatoda*	Leaves	ZnO	Antibacterial	[79]
*Hydnocarpus alpinus*	Root	ZnO	Photocatalytic	[80]
*Aristolochia indica*	Root	ZnO	Antimicrobial	[81]
*Euphorbia heterophylla*	Leaves	ZnO	Antibacterial	[83]
*Aeromonas hydrophila*	Plant	ZnO	Antifungal	[84]
*Mirabilis jalapa*	Leaves	ZnO	Antioxidant	[85]
*Trianthema portulacastrum*	Flower	ZnO	Photocatalytic	[86]
*Trifolium pratense*	Peel	ZnO	Antibacterial	[87]
*Punica granatum*	Leaves	ZnO	Cytotoxicity	[88]
*Medicago sativa*	Leaves	ZnO	Cytotoxicity	[89]
*Cucurbita pepo*	Leaves	ZnO	Photocatalytic	[90]
*Costus woodsonii*	Leaves	ZnO	-	[91]
*Raphanus sativus*	Root	ZnO	Antimicrobial	[92]
*Garcinia xanthochymus*	Plant	ZnO	Photocatalytic	[93]
*Tecoma castanifolia*	Leaves	ZnO	Anticancer	[94]
*Pongamia pinnata*	Leaves	ZnO	Antibacterial	[95]
*Ruta chalepensis*	Leaves	ZnO	Photocatalytic	[96]
*Coriandrum sativum*	Leaves	ZnO	Antimicrobial	[97]
*Thymus vulgaris*	Leaves	ZnO	Photocatalytic	[98]

**Table 3:** Synthesis of silver, copper, cadmium, and magnesium NPs from biological sources and applications.

Name of material	Source	Nanoparticles	Applications	Reference
*Allium ampeloprasum*	Leaves	Silver	Antiinflammatory	[99]
*Crataegus pentagyna*	Fruit	Silver	Photocatalytic degradation	[100]
*Crataegus microphylla*	Fruit	Silver	Antibacterial	[101]
*Annona muricata*	Leaves	Silver	Anticancer	[102]
*Handroanthus heptaphyllus*	Leaves	Silver	-	[103]
*Tamarindus indica*	Fruit	Silver	Anticancer	[104]
*Andrographis paniculata*	Leaves	Silver	In vitro antifilarial	[105]
*Fumaria parviflora*	Plant	Silver	Cytotoxicity	[108]
*Delonix regia*	Leaves	Silver	In vitro cytotoxicity	[109]
*Calotropis gigantea*	Leaves	Silver	Larvicidal	[110]
*Portieria hornemannii*	Algae	Silver	Antibacterial	[111]
*Gelidium corneum*	Algae	Silver	Antimicrobial	[112]
*Botryococcus braunii*	Algae	Silver	-	[113]
Jackfruit	Seed	Silver	Antimicrobial	[115]
*Combretum erythrophyllum*	Leaves	Silver	Antibacterial	[116]
*Allium cepa*	Plant	Silver	Antidiabetic	[117]
*Laminaria japonica*	Algae	Silver	-	[118]
*Ampelocissus latifolia*	Leaves	Silver	Antibacterial	[119]
*Rhododendron ponticum*	Leave	Silver	Antibiofilm	[120]
*Nauclea latifolia*	Fruit	Silver	Antioxidant	[121]
*Annona reticulata*	Leaves	Silver	Antimicrobial	[122]
*Tinospora crispa*	Leaves	Copper	-	[124]
*Thymus vulgaris*	Leaves	Copper	Catalytic	[125]
*Melissa officinalis*	Leaves	Copper	Antibacterial activity	[126]
*Opuntia ficus-indica*	Fruit	Cadmium	Solar cell	[127]
Turmeric	Plant	Cadmium	Antibacterial	[128]
Olive	Leaves	Cadmium	Antifungal	[129]
*Penicillium chrysogenum*	Fungus	Magnesium	Antimicrobial	[130]
*Bauhinia purpurea*	Leaves	Magnesium	Antibacterial	[131]
*Turbinaria ornata*	Algae	Magnesium	Antimycobacterial	[132]
*Sargassum wightii*	Algae	Magnesium	Antifungal	[133]
*Withania somnifera*	Leaves	Magnesium	Antifungal	[134]
*Pisonia alba*	Leaves	Magnesium	Antimicrobial	[135]

*Psidium guajava*	Leaves	Copper	Photocatalytic	[136]
